# Single-cell profiling of lncRNAs in the developing human brain

**DOI:** 10.1186/s13059-016-0933-0

**Published:** 2016-04-14

**Authors:** Qing Ma, Howard Y. Chang

**Affiliations:** Center for Personal Dynamic Regulomes and Program in Epithelial Biology, Stanford University School of Medicine, Stanford, CA 94305 USA

## Abstract

Single-cell RNA-seq in samples from the human neocortex demonstrate that long noncoding RNAs (lncRNAs) are abundantly expressed in specific individual brain cells, despite being hard to detect in bulk samples. This result suggests that the lncRNAs might have important functions in specific cell types in the brain.

Please see related Research article: www.dx.doi.org/10.1186/s13059-016-0932-1

## Introduction

A large variety of long noncoding RNAs (lncRNAs) are expressed in brain tissue and are emerging as key regulators of neuronal function and diseases [1]. Previous findings have suggested that lncRNA expression is more tissue and cell-type specific than mRNA expression [2], leading to the possibility that lncRNAs could be key regulators of cell fate and cell-type-specific function. The human brain has dramatic complexity generated across various different cell types, and previous studies have suggested subtype-dependent enrichment of lncRNAs in the human cortex [3]. Now, Liu and colleagues have deployed single-cell RNA-seq of the human brain to provide a greater resolution of cell-type and single-cell specificity for lncRNAs [4].

## Transcriptome reference of lncRNAs in human brain development

The majority of the human genome is transcribed to produce lncRNAs. Many of these are believed to play important roles in regulating neuronal development, brain functions and neuronal diseases. The brain produces a large variety of lncRNAs, but, when estimated by analysis of bulk tissue, the expression levels of lncRNAs are on average lower compared with those of mRNAs. It is possible that lncRNAs are expressed at uniformly low levels in all cells or it is plausible that they are more highly expressed and functional in only a subset of cells, and this is masked in bulk studies. Some previous reports have suggested the latter explanation of single-cell specificity for lncRNA expression in the immune system and the brain [3, 5–7]. In this issue, Liu and colleagues report the effect of combining bulk human brain RNA-seq and single-cell RNA-seq to further profile temporal and cell-type-specific lncRNA expression during development of the neocortex [4].

Previous annotations of lncRNAs in human brain were based on polyadenylated (polyA) transcript selection and RNA-seq library preparations that did not preserve strand information. As a result, the non-polyA and antisense lncRNAs, some of which have been shown to have important functions, were dismissed. In order to get a more comprehensive annotation and quantification of lncRNAs, the authors used bulk brain tissue and performed strand-specific RNA-seq of both polyA-selected RNA and rRNA-depleted total RNA. To identify temporal lncRNA expression during human neocortical development, samples and data were collected at four developmental time-points (gestational weeks 13/14.5, 16, 21 and 23). Through this methodology, a large number of novel lncRNAs were annotated, including some antisense lncRNAs and non-polyA lncRNAs. The lncRNAs and mRNAs that were differentially expressed across the four developmental time-points were also identified. Thus, this more-comprehensive lncRNA transcriptome serves as a better reference for single-cell RNA-seq analysis and lncRNA profiling during brain development.

## Abundant lncRNA expression in a subpopulation of single cells

In bulk samples, mRNAs are expressed on average 13.6-fold higher than lncRNAs [4]. To determine whether lncRNAs are expressed highly in subpopulations of cells, the authors captured single cells from different stages of development of the neocortex for subsequent RNA-seq. Based on RNA-seq data of 276 single cells, the authors analyzed the abundance of lncRNAs by comparing the median expression of lncRNAs with the median expression of mRNAs (lncRNA:mRNA median ratios). The bulk samples had a lncRNA:mRNA ratio as low as 0.31, whereas the single cells had a ratio of approximately 0.85, with 32.2 % of cells exceeding 1.0. This result suggests that lncRNAs are expressed at levels comparable with those of mRNAs in individual cells in human neocortex, in contrast to their being detected at low levels in bulk brain samples. Consistent with this hypothesis, after clustering single cells with known cell-type-specific markers, lncRNAs were detected in fewer cells than mRNAs, and lncRNAs have greater cell-type specificity. In addition, lncRNAs that were detected at low levels in bulk samples were expressed in fewer single cells and were more cell-type specific than higher-abundance lncRNAs. To further validate the cell-type-specific expression of lncRNAs, the authors also performed in situ hybridization for three lncRNAs and confirmed their expression pattern. Among those three lncRNAs, one of the radial glia-specific lncRNAs—*LOC646329*—was found to regulate cell proliferation when assessed by knockdown mediated by clustered regularly interspersed palindromic repeats interference (CRISPRi). Taken together, the authors demonstrate that many lncRNAs are abundantly expressed in individual cells, and these lncRNAs might have important cell-type-specific functions in human brain.

## Concluding remarks

Gene expression from bulk-sample RNA-seq can be modeled as an output related to both the expression levels of genes in each cell type and the relative abundance of each cell type. Given that many lncRNAs are expressed only in certain cell types, various crucial lncRNA-related phenomena might be either invisible or only partially characterized when average data across a bulk population of cells are analyzed. Not only at the cell-type level, expression and fluctuation of lncRNAs at the individual cell level might also control cell fate, cell function and cell communication. Therefore, single-cell transcriptome analysis provides a new dimension and a higher resolution for identifying and studying lncRNA functions. In addition, the human neocortex lncRNA reference catalog might provide information concerning lncRNAs that can serve as cell-type-specific markers as they are even more specific compared with coding genes in certain cell types. However, the potential problems for this study are that not all the cell types are captured, and thus certain cell types might be preferentially enriched. Also, the number of lncRNAs detected in single-cell RNA-seq (1400) are much lower compared with those from bulk-sample RNA-seq (11,642). Information from other studies in mouse and human brain would be helpful to better understand the relative abundances and features of different cell types in the developing human brain. Finally, a larger-scale study to sequence more single cells might help to increase the coverage. Thus, recent advances in single-cell ‘omics’ are providing unprecedented opportunities to study the complexities of various biological systems at ever-higher resolution.

## Abbreviations

CRISPRi: clustered regularly interspersed palindromic repeats interference
lncRNA: long non-coding RNA
polyA: polyadenylated
